# Quantification of population benefit in evaluation of biomarkers: practical implications for disease detection and prevention

**DOI:** 10.1186/1472-6947-14-15

**Published:** 2014-03-06

**Authors:** Xiaohong Li, Patricia L Blount, Brian J Reid, Thomas L Vaughan

**Affiliations:** 1 Divisions of Public Health Sciences, Fred Hutchinson Cancer Research Center, 1100 Fairview Ave. N., Seattle, WA 98109, USA; 2 Human Biology, Fred Hutchinson Cancer Research Center, 1100 Fairview Ave. N., Seattle, WA 98109, USA; 3 Department of Medicine, University of Washington, 1959 NE Pacific Street, Seattle, WA 98195, USA; 4 Department of Epidemiology, University of Washington, 1959 NE Pacific Street, Seattle, WA 98195, USA; 5 Department of Genome Sciences, University of Washington, 1959 NE Pacific Street, Seattle, WA 98195, USA

**Keywords:** Ratio of population benefit, RPB, Biomarkers, Disease prevention, Disease early detection, Clinical decision making, Biomarkers for early detection, Risk/benefit analysis

## Abstract

**Background:**

With the rapid development of “-omic” technologies, an increasing number of purported biomarkers have been identified for cancer and other diseases. The process of identifying those that are most promising and validating them for use at the population level for prevention and early detection is a critical next step in achieving significant health benefits.

**Methods:**

In this paper, we propose that in order to effectively translate biomarkers for practical clinical use, it is important to distinguish and quantify the differences between the use of biomarkers and other risk factors to identify preventive interventions versus their use in disease risk prediction and early detection. We developed mathematical models for quantitatively evaluating risk and benefit in use of biomarkers for disease prevention or early detection. Simple numerical examples were used to demonstrate the potential applications of the models for various types of data.

**Results:**

We propose an index which takes into account potential adverse consequences of biomarker-driven interventions – the ‘naïve’ ratio of population benefit (RPB) – to facilitate evaluating the potential impact of biomarkers on cancer prevention and personalized medicine. The index RPB is developed for both binary and continuous biomarkers/risk factors. Examples with computational analyses are presented in the paper to contrast the differences in using biomarkers/risk factors for prevention and early detection.

**Conclusions:**

Integrating epidemiologic knowledge into clinical decision making is a key step to translate new biomarkers/risk factors into practical use to achieve health benefits. The RPB proposed in this paper considers the absolute risk of a disease in intervention, and takes into account the risk-benefit effects simultaneously for a marker/exposure at the population level. The RPB illustrates a unique approach to quantitatively assess the risk and potential benefits of using a biomarker/risk factor for intervention in both early detection and prevention.

## Background

The identification of robust cancer risk factors and biomarkers are the cornerstones of modern approaches to cancer prevention and personalized medicine. A large number of environmental and host risk factors (either inherited or somatic) have been identified that are associated with cancer risk, and with rapidly-advancing “-omics” technologies, the reported number of biomarkers proposed for clinical use is increasing dramatically. However, the translation of these for use in the population or clinic in such a way as to have a significant impact on cancer incidence and mortality is still a major challenge. The process of selecting and evaluating the most promising biomarkers for clinical application among the large number of purported biomarkers is a critical step in the translation process. Key to this process is distinguishing the differences between evaluating biomarkers and risk factors for primary prevention programs *versus* disease risk prediction and early detection. Quantitative analysis of these differences can facilitate the translational process.

Pepe *et al.*[1] compared the association of a marker with a disease, often quantified in case-control or cross-sectional studies by the odds ratio (OR), with use of the marker for disease classification (i.e. presence or absence of cancer in a sample), and illustrated the limitation of the OR in gauging the performance of a diagnostic, prognostic, or screening marker. More recently, the use of markers discovered in genetic association studies for disease risk prediction was specifically addressed by Jakobsdottir *et al*. [2]. The limitations of using markers for medical diagnosis or early detection also have been comprehensively assessed [3-5]. Recently, an increasing number of studies have focused on the use of previously-identified risk factors and biomarkers (e.g., one or more constitutive SNPs (single-nucleotide polymorphism)) for cancer prevention or for pathway-targeted therapy development [6,7]. In practice, the specific criteria for evaluating a biomarker or risk factor for disease detection/prediction could be quite different than that for disease prevention. To highlight and evaluate these differences quantitatively, we first illustrate the numerical relationship between the OR of a biomarker/risk factor and its population attributable risk percent (PAR%) (assuming causality) in the context of a population prevention program. We then illustrate the corresponding accuracy, as measured by sensitivity and specificity, for a biomarker/risk factor with identical characteristics (OR and prevalence) in the context of a disease detection/prediction program. Finally we propose an index – the ‘naïve’ ratio of population benefit (RPB) – for quantifying overall risk/benefit of using a biomarker for cancer prevention or detection/prediction at the population level. Analyses are presented separately for binary and continuous biomarkers.

## Methods and results

### Numerical relationships between sensitivity, specificity and population attributable risk for binary and continuous biomarkers

#### Calculation for binary marker/risk factor

The PAR% is often used to estimate the fraction of the total disease burden in the population that would not have occurred if a causal risk factor were absent [8]. To help introduce the latter parts of the paper, we first illustrate the numerical relationships between PAR% for causal binary markers/risk factors at different prevalence and relative risk levels and the corresponding sensitivity and specificity of using a marker/risk factor with identical characteristics for disease classification/prediction (Table 1). The status of a specific binary risk factor (e.g. a mutated gene or exposure) and the observed disease outcome status, also binary, can be displayed as a standard 2 × 2 contingency table, with the four cells labeled *a*(+/+), *b*(+/-), *c*(-/+) and *d*(-/-) corresponding to the counts of individuals in a cohort with status of exposure and outcome (+ for yes, - for no), respectively. If outcome is directly predicted by the marker, for calculation of sensitivity and specificity (either for screening or screening-based disease intervention), the data can be arranged in an identical 2 × 2 table with the individual cells labeled *a* (true positive), *b* (false positive), *c* (false negative) and *d* (true negative) relating the biomarker status with a true outcome status or “gold standard”.

**Table 1:** Numerical illustration for calculating RPB of hypothetical binary markers using data of three cancers as examples

**Biomarker/exposure characteristics**		**Ratio of population benefit (RPB)**						**Net benefit (NB)**		
**OR**	**Prevalence (%)**	**PAR%**	**Sen.**	**Spe.**	**RPB**	**RPB**	**RPB**	**NB**	**NB**	**NB**
					**(EA, *f *** **0.03)**	**(Breast ca, *f *** **0.06)**	**(Ovarian ca, *f *** **0.15)**	**(EA, *f *** **0.03)**	**(Breast ca, *f *** **0.06)**	**(Ovarian ca, *f *** **0.15)**
1.5	0.1	0.05	0.001	0.999	-0.000	-0.001	-0.001	-0.000	-0.000	-0.000
2	0.1	0.10	0.002	0.999	-0.000	-0.001	-0.001	-0.000	-0.000	-0.000
4	0.1	0.29	0.004	0.999	0.000	-0.000	-0.001	0.000	-0.000	-0.000
10	0.1	0.81	0.009	0.999	0.002	0.001	-0.000	0.000	0.000	-0.000
20	0.1	1.56	0.017	0.999	0.004	0.002	0.000	0.000	0.000	0.000
50	0.1	3.19	0.033	0.999	0.008	0.004	0.001	0.000	0.000	0.000
1.5	1	0.49	0.015	0.990	-0.004	-0.006	-0.008	-0.000	-0.000	-0.001
2	1	0.97	0.020	0.990	-0.002	-0.006	-0.008	0.000	-0.000	-0.001
4	1	2.81	0.038	0.990	0.002	-0.003	-0.007	0.000	-0.000	-0.001
10	1	7.61	0.085	0.991	0.015	0.004	-0.003	0.001	0.000	-0.001
20	1	13.93	0.148	0.991	0.031	0.014	0.001	0.001	0.001	0.000
50	1	26.33	0.271	0.993	0.063	0.033	0.010	0.002	0.002	0.002
1.5	10	4.70	0.142	0.900	-0.039	-0.065	-0.084	-0.002	-0.004	-0.013
2	10	8.94	0.180	0.901	-0.029	-0.059	-0.082	-0.001	-0.004	-0.013
4	10	22.54	0.303	0.902	0.003	-0.040	-0.073	0.000	-0.003	-0.012
10	10	46.05	0.514	0.904	0.058	-0.008	-0.057	0.002	-0.001	-0.009
20	10	63.92	0.675	0.906	0.100	0.017	-0.046	0.004	0.001	-0.007
50	10	81.79	0.836	0.907	0.141	0.041	-0.034	0.006	0.003	-0.005
1.5	30	12.90	0.390	0.701	-0.125	-0.200	-0.256	-0.005	-0.014	-0.041
2	30	22.80	0.460	0.702	-0.107	-0.189	-0.251	-0.004	-0.013	-0.040
4	30	46.84	0.628	0.703	-0.064	-0.163	-0.238	-0.003	-0.011	-0.038
10	30	72.43	0.807	0.705	-0.017	-0.136	-0.225	-0.001	-0.009	-0.036
20	30	84.69	0.893	0.706	0.005	-0.123	-0.219	0.000	-0.009	-0.035
50	30	93.44	0.954	0.707	0.021	-0.114	-0.215	0.001	-0.008	-0.034
1.5	70	25.71	0.777	0.301	-0.327	-0.486	-0.606	-0.013	-0.034	-0.096
2	70	40.89	0.823	0.301	-0.316	-0.480	-0.603	-0.013	-0.033	-0.096
4	70	67.46	0.902	0.302	-0.295	-0.467	-0.597	-0.012	-0.032	-0.095
10	70	86.14	0.958	0.303	-0.280	-0.459	-0.593	-0.011	-0.032	-0.094
20	70	92.92	0.979	0.303	-0.275	-0.456	-0.591	-0.011	-0.032	-0.094
50	70	97.13	0.991	0.303	-0.272	-0.454	-0.591	-0.011	-0.031	-0.094

ca= cancer. EA= esophageal adenocarcinoma. f= loss adjustment factor of quality-adjusted life year. NB= net benefit. OR= odds ratio.

The table shows numerical relationship between Odds ratio, Marker prevalence, PAR% of binary markers and their RPB based on the cancer data of three studies. NB were also calculated.

The table assumes 1% disease prevalence in general population. For PAR%, sensitivity and specificity similar patterns are observed with other disease prevalence values less than about 10%. Disease prevalence affects RPB more directly (see text).

Using the counts in the four cells of the contingency table (whether corresponding to exposure and outcome or disease classification) several commonly used quantities can be obtained. A binary marker/risk factor has two possible values, leading to fixed sensitivity (*a*/(*a + c*)) and specificity (*d*/(*b + d*)) values in a population with a specific OR and marker prevalence, in which the incidences in exposed (or marker carriers) and unexposed (or marker non-carriers) are a/(a + b), c/(c + d) respectively. Table 1 shows the numerical relationships among i) prevalence of a risk factor/marker, ii) the relative risk of disease associated with the marker (indicated by OR), iii) PAR%, iv) sensitivity, and v) specificity. If we let *r*_*1*_ = ((*a + c*)/(*a + b + c + d*)), *r*_*2*_ = *c*/(*c + d*), (*r*_*1*_ is the prevalence and *r*_*2*_ is the false negative fraction), the population attributable risk can be calculated as PAR% = (*r*_*1*_*- r*_*2*_)/*r*_*1*_100%. By definition, the false positive fraction = 1-specificity; the false negative fraction = 1-sensitivity; and the OR = (sensitivity/(1-sensitivity))/((1-specificity)/specificity). In Table 1, marker prevalence and risk factor exposure prevalence are interchangeable algebraically; the former used for early detection and risk prediction, and the later used for prevention. The calculation of PAR% above was based on the assumption of no adjustment for potential confounders. A common way to obtain PAR% adjusted for confounders is to use a stratification approach: $\mathrm{PA}R_{\mathrm{adj}}\%=\sum_{i}p_{i}\mathrm{PA}R_{i}\%$ where *p*_*i*_ is the proportion of cases in stratum *i*, PAR_i_% is the PAR% estimated from stratum *i*. More details for dealing with confounders can be found in Rothman *et al*. [9].

#### Numerical analysis for continuous marker/risk factor

**Figure 1:**
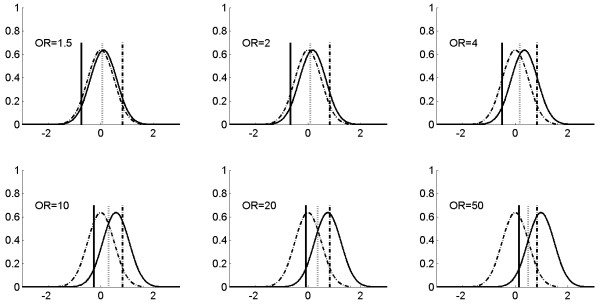
**Hypothetical distribution patterns of continuous markers with different relative risks, and thresholds for risk prediction.** Two normal distributions (mean = 0 and standard deviation = 0.5) are used to represent the distribution of a continuous marker in disease (solid curved line) and non-disease (dashed curved line) populations for six different ORs. The locations of the means for the disease population are consistent with the logit model *Pr*(*D =* 1*|X*) = *α + βX*, in which one unit increase corresponds to the OR shown in the figure. The three vertical bars (solid, dotted, and dashed) correspond to different thresholds (cut off value ‘*c*’) for positive-negative calls of a disease with a continuous distribution marker. Specifically, the solid bar represents the threshold value *c* such that the sensitivity is kept for 0.95 for various OR values in the plot; the dotted bar represents the threshold value *c* such that the sensitivity and specificity are equal for various OR values in the plots; and the dashed bar represents the threshold value *c* such that the specificity is kept for 0.95 for various OR values in the plot. The examples of using the continuous marker for disease classification or prevention are shown in Figure 2; and corresponding sensitivity, specificity and PAR% of various thresholds (three bars in this Figure) are shown in Table 2 and Figure 2 (the cross, circle, and triangle in Figure 2 correspond to solid, dashed, and solid vertical bars in Figure 1).

Pepe *et al*. [1] evaluated the limitation of the OR in gauging the performance of a diagnostic, prognostic, or screening marker. Illustrated here is the use of continuous biomarkers both for diagnostic/prognostic/screening and for prevention, along with the relationship between the OR value and PAR% parameters for the continuous markers. Figure 1 presents a few hypothetical normal distributions of continuous markers/risk factors with different OR risk values.

For the continuous distribution markers, the sensitivity and specificity can be calculated as follows [10], $\mathrm{Sensitivity}=P\left(Y_{D}>c\right)=\Phi \left(\frac{\mu_{D}-c}{\sigma_{D}}\right)$; $\mathrm{Specificity}=1-P\left(Y_{\bar{D}}>c\right)=1-\Phi \left(\frac{\mu_{\bar{D}}-c}{\sigma_{\bar{D}}}\right)$, where $\bar{D}$ indicates non-disease group, and *c* is the threshold above which a positive (disease) call will be made. In contrast to binary markers, which only have one set of sensitivity and specificity values, continuous markers can be used to generate infinite sets of sensitivity and specificity values depending on the threshold value of *c*.

**Figure 2:**
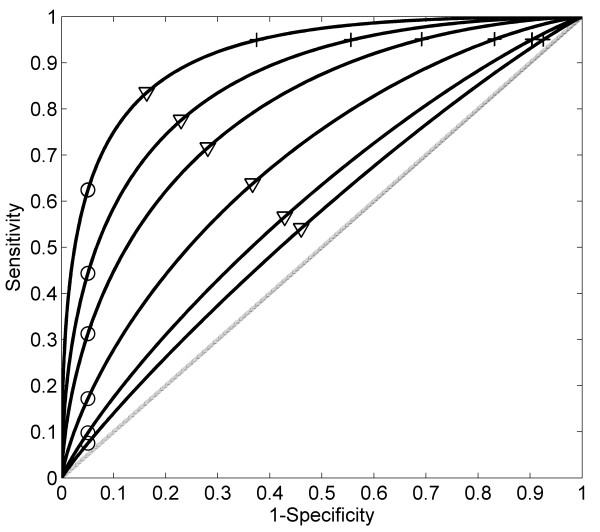
**Disease prediction performance evaluated by ROC curves for the hypothetical continuous markers with different relative risks.** ROC curves for continuous risk marker with different odds ratios (from bottom to top OR = 1.5, 2, 4, 10, 20, 50), which corresponding to the distribution plots of continuous markers shown in Figure 1. The crosses correspond to a fixed sensitivities; the circles to a fixed specificities; and triangles to equal sensitivities and specificities. Their corresponding PAR% values are shown in Table 2.

To quantify PAR% for continuous markers, let *w* be the proportion of diseased individuals in a population or risk of a disease in the general population, then for a marker with a continuous value, a specific set of sensitivity and specificity is obtained for a given threshold *c*, the risk of ‘unexposed’ (the proportion of subjects, either diseased or non-diseased whose marker level is lower than the threshold *c*) *q*_*ue*_ with threshold *c* can be calculated as $q_{\mathrm{ue}}=\frac{w\left[1-\int_{c}^{\infty }f_{d}\left(x\right)\mathrm{dx}\right]}{\left(1-w\right)\int_{-\infty }^{c}f_{\bar{d}}\left(x\right)\mathrm{dx}+w\left[1-\int_{c}^{\infty }f_{d}\left(x\right)\mathrm{dx}\right]}=\frac{w\left(1-\mathrm{sensitivit}y_{c}\right)}{\left(1-w\right)\mathrm{specificit}y_{c}+w\left(1-\mathrm{sensitivit}y_{c}\right)}$, where *f*_*d*_(*x*) and $f_{\bar{d}}\left(x\right)$ are the probability density distribution of a biomarker in the diseased and non-diseased group respectively (assuming normal distribution), *sensitivity*_*c*_ and *specificity*_*c*_ are the sensitivity and specificity of the continuous marker at threshold *c*. Therefore, for a continuous marker, we have PAR% = (*w* – *q*_*ue*_)/*w*. Table 2 shows the numerical relationships among sensitivity and specificity and PAR% of the quantification for various thresholds *c* in Figure 2.

**Table 2:** Numerical illustration for calculating RPB of hypothetical continuous markers using data of three cancers as examples

**Biomarker/exposure characteristics**^**a**^					**Ratio of population benefit (RPB)**			**Net benefit (NB)**		
**(Threshold)**^**b**^	**OR**	**PAR%**	**Sen.**	**Spe.**	**RPB**	**RPB**	**RPB**	**NB**	**NB**	**NB**
					**(EA, f 0.03)**	**(Breast ca, f 0.06)**	**(Ovarian ca, f=0.15)**	**(EA, f=0.03)**	**(Breast ca, f=0.06)**	**(Ovarian ca, f=0.15)**
Fixed sensitivity (95%)	1.5	33.22	0.95	0.07	-0.456	-0.659	-0.812	-0.018	-0.046	-0.129
	2	48.88	0.95	0.09	-0.440	-0.641	-0.792	-0.017	-0.045	-0.126
	4	71.30	0.95	0.16	-0.389	-0.583	-0.728	-0.015	-0.040	-0.115
	10	84.46	0.95	0.29	-0.287	-0.466	-0.601	-0.011	-0.032	-0.095
	20	89.01	0.95	0.43	-0.187	-0.352	-0.475	-0.007	-0.024	-0.075
	50	92.25	0.95	0.61	-0.051	-0.196	-0.305	-0.002	-0.014	-0.048
Fixed specificity (95%)	1.5	2.59	0.08	0.95	-0.019	-0.032	-0.043	-0.001	-0.002	-0.007
	2	4.95	0.10	0.95	-0.013	-0.029	-0.041	-0.001	-0.002	-0.007
	4	12.67	0.17	0.95	0.006	-0.018	-0.036	0.000	-0.001	-0.006
	10	27.40	0.31	0.95	0.041	0.002	-0.028	0.002	0.000	-0.004
	20	41.15	0.44	0.95	0.074	0.021	-0.019	0.003	0.001	-0.003
	50	60.16	0.62	0.95	0.119	0.047	-0.008	0.005	0.003	-0.001
Balanced sensitivity & specificity^c^	1.5	14.83	0.54	0.54	-0.208	-0.316	-0.397	-0.008	-0.022	-0.063
	2	23.89	0.57	0.57	-0.178	-0.285	-0.366	-0.007	-0.020	-0.058
	4	42.57	0.64	0.63	-0.114	-0.222	-0.304	-0.005	-0.015	-0.048
	10	60.28	0.72	0.72	-0.030	-0.137	-0.218	-0.001	-0.010	-0.035
	20	70.68	0.78	0.77	0.024	-0.085	-0.166	0.001	-0.006	-0.026
	50	80.17	0.84	0.84	0.088	-0.020	-0.101	0.003	-0.001	-0.016

Numerical relationships between Odds ratio and PAR% of continuous markers and their RPB based on the data of three studies.

ca= cancer. EA= esophageal adenocarcinoma. f = loss-adjustment factor of quality-adjusted life year. NB= net benefit.

OR= odds ratio. Sen.= sensitivity, Spe.= specificity.

The disease prevalence of population used for the table = 0.01. As shown in the formula of RPB for continuous markers, disease prevalence w will directly affect the value of RPB.

^a^Hypothetical continuous biomarker/exposure with assumed distributions as described in Figure 1.

^b^Threshold used for continuous biomarkers positive are the thresholds shown in Figure 1 (three vertical bars).

^c^Using a threshold that lead to sensitivity and specificity closest to the upper left corner of ROC curve coordinates for cutoff.

#### Distinguishing the use of biomarkers/risk factors for cancer detection and prevention

Above we presented the numerical relationships between sensitivity, specificity and PAR% for binary and continuous biomarkers (Tables 1 and 2). Below we use examples to illustrate the importance of distinguishing between the use of biomarkers for cancer detection/risk prediction and for cancer prevention since the consequences of false positive and false negative findings may differ substantially in these two contexts.

*Example 1: Genotype and bladder cancer.* A genetic association study [11] showed strong evidence that the copy number of gene GSTM1 is significantly associated with risk of bladder cancer, with an OR = 1.9 corresponding to the GSTM1 null genotype (51% prevalence). If this marker were used as a binary marker for bladder cancer detection in the general population, it would result in 66% sensitivity and 50% specificity, a poor marker for diagnostic purposes. However, if a drug were to be developed that targeted the pathway(s) by which GSTM1 null increases risk, and if the drug were 100% effective in preventing bladder cancer without toxic side effects (and ignoring costs), then treatment of all marker carriers would reduce bladder cancer by 31% (PAR%), which would represent a substantial public health benefit. One way to quantify such a benefit can be performed using the method developed in this paper as shown in example 4.

*Example 2: Smoking and lung cancer.* Using Table 1, if the prevalence of smoking (risk factor) in a population is 30%, and the OR of smoking for lung cancer risk is estimated to be 10- to 20-fold higher than the non-smokers, then the corresponding PAR% value is 73-85% (had all smokers not smoked, there would have been 73% to 85% fewer lung cancers). The corresponding false positive fraction is about 29.3%, which indicates among the non-lung cancer group (normal), 29.3% are smokers. This high ‘false positive’ fraction may be tolerable for lung cancer prevention since reducing 73-85% of lung cancers at the ‘expense’ of abstaining from smoking is likely acceptable. (If other diseases caused by smoking are considered, this argument is even stronger). Quantification of such benefit can be accomplished using the method developed in this paper as shown in example 3.

### Quantitative evaluation of the benefit of using biomarkers for disease detection/prediction and disease prevention at the population level

The above numerical analyses and specific examples indicate that traditional measures of association (OR, PAR%, sensitivity, specificity, and others) can have dramatically different implications depending on whether they are applied to risk prediction, early detection or prevention of disease. Since false positive and false negative classifications are unavoidable in practice [12], we propose an index, the ‘naïve’ ratio of population benefit (RPB), which takes into account the adverse effects of misclassification, for evaluating the impact of using biomarkers for early detection/risk prediction and preventive interventions on a disease at the population level. Unlike OR, PAR%, sensitivity, specificity, and other similar measures which do not directly depend on disease prevalence, the RPB does account for disease prevalence in a reasonable way as shown in the following parts of the paper. This new index is not intended for evaluating or comparing the prediction accuracy of biomarkers or prediction models; instead it is intended for analyzing the potential benefit for a population using a previously-selected biomarker for disease intervention after taking into account potential adverse effects.

#### RPB for binary markers/risk factors

Using the 2 × 2 contingency table introduced earlier, if no biomarker is used for early cancer detection/cancer risk prediction, lethal cancer cases will occur (*a* + *c*); with a subset of individuals (*b* + *d* ) remaining cancer free. The quantification of lives lost in this situation is - *f*_1_ _∗_ (*a* + *c*), and lives gained is *f*_2_ _∗_ (*b* + *d*), with the negative sign indicating loss; (- *f*_1_ represents naïve quantification of lives lost due to cancer cases discovered at a late incurable stage), a positive value, *f*_1_ represents lives gained if cancer is detected early, and *f*_2_ represents naïve quantification of lives gained due to non-cancer subjects who are not classified as cancer (gain due to perfect markers with no false positives and - *f*_2_ represents loss due to a false positive call). If a binary biomarker is used for cancer detection, then let *a* be the cancer cases that will be detected earlier (true positive), and *b* be the number of non-cancer cases are classified as cancer due to false positives associated with this biomarker. The sum of gains and losses associated with this biomarker is *f*_1_*a* + (-*f*_1_*c*) + (-*f*_2_*b*) + *f*_2_*d*. Note, the sum of losses and gains associated with not using the biomarker - *f*_1_(*a* + *c*) + *f*_2_(*b* + *d*); hence, the change of total net gain for comparison using a biomarker vs. no biomarker is *f*_1_*a* - *f*_2_*b,* (no change for - *f*_1_*c* + *f*_2_*d* when comparing the two sums, assuming false negative calls *c* will be treated the same as no biomarker). For a population, if all cancer cases could be detected early without false positives (an ideal marker), the sum of gains and losses for the population is *f*_1_ _∗_ (*a* + *c*) + *f*_2_ _∗_ (*b* + *d*). Therefore, assuming binary biomarkers for cancer detection are not perfect (with false positives and false negatives), a naïve estimation of the ratio of population benefit (RPB) can be estimated by $\mathrm{RPB}=\frac{f_{1}a-f_{2}b}{f_{1}\left(a+c\right)+f_{2}\left(b+d\right)}=\frac{af_{1}-bf_{2}}{af_{1}+bf_{2}+cf_{1}+df_{2}}$. Note that changes in disease prevalence are accounted for in the RPB calculation, which uses all of the terms defining prevalence (*a + c*)/(*a + b + c + d*). The RPB is different from Net Benefit (NB) based on decision curve analysis [13,14]. NB = *(a-wb)/(a + b + c + d),* where *w* is the weight for counting the cost of false positive relatives to the cost of false negatives. The denominator of NB counts the cost of the overall population, whereas the denominator of RPB only counts the cost for worst possible performance of a marker i.e. the counts for true positive *a* and true negative *d* both are 0 in prediction. In addition, RPB also considers adverse effect (*δ*) due to intervention as shown in the next section. Therefore, RPB is more sensitive in evaluating false positive or false negative costs compare to NB. The adjusted RPB for potential confounders can also be obtained by the weighted average of individual RPB for each strata in stratified analysis, a similar idea to the adjusted PAR% mentioned above [9]. $\mathrm{RP}B_{\mathrm{adj}}=\sum_{i}p_{i}\frac{a_{i}{f_{1}}_{i}-b_{i}{f_{2}}_{i}}{a_{i}f_{1i}+b_{i}{f_{2}}_{i}+c_{i}f_{1i}+d_{i}f_{2i}}$, where *p*_*i*_ is the proportion of cases in stratum *i*, and *a*_*i*_, *b*_*i*_, *c*_*i*_, *d*_*i*_, *f*_1*i*_, and *f*_2*i*_ are same as in RPB above but are estimated from specific stratum *i.* RPB is a percentage of net gain, which is a ratio of net gains in the group of marker carriers (or risk factor exposed), including diseased and non-diseased, against overall gain estimated by quantifying the losses and gains due to false positive, false negative, true positive and true negative. Disease prevalence is considered in RPB calculation.

For cancer prevention with consideration of adverse effects (i.e. prevention measure is applied to the carriers of a predictive (or risk-causal) biomarker or those exposed to a risk factor), the $\mathrm{RPB}=\frac{\mathrm{a\eta}f_{1}-\mathrm{a\delta}-\mathrm{b\delta}}{af_{1}+bf_{2}+cf_{1}+df_{2}}$, similarly, *f*_1_ and *f*_2_ are defined as the same as the binary marker mentioned above; *η* represents the efficacy of a prevention measure (i.e. the percentage of cancer reduced due to a prevention measure); and *δ* represents possible adverse effect of a prevention measure (i.e. side effect of a drug for cancer prevention). When there is no adverse effect from a prevention measure, *δ* = 0. Numerically, RPB could be negative, 0 or positive, which indicates detrimental, neutral, or beneficial overall effects at the population level, respectively. In addition, the absolute gain for early cancer detection or cancer prevention may be quantified as (*af*_*1*_*- bf*_*2*_) – *h*(*a + b*), and (*aηf*_*1*_*- aδ - bδ*) – *h*(*a + b*) respectively, where *h* is the coefficient for the cost of prevention or treatment of exposed subjects or subjects positive for specific markers. Following are two examples illustrating the use of RPB for binary markers and risk factors.

*Example 3: Smoking and lung cancer.* Since there are no negative health effects due to abstaining from smoking, we set *δ* = 0, and RPB becomes *aηf*_*1*_/(*af*_*1*_*+ bf*_*2*_ *+ cf*_*1*_*+ df*_*2*_), where *η* in the example represents efficacy (%) of lung cancer reduction due to abstaining from smoking.

*Example 4: Genotype and bladder cancer*. GSTM1 is a bladder cancer associated biomarker (marker prevalence = 51%, OR = 1.9). If a drug were developed that targeted the effect associated with the null GSTM1 variant, and if all carriers of the risk variant were treated with the drug, the drug had no adverse side effects and is 100% effective (*δ* = 0, *η = 1*), then RPB = *af*_*1*_/(*af*_*1*_*+ bf*_*2*_ *+ cf*_*1*_*+ df*_*2*_). However, if the efficacy of a drug *η* is much less than 1 and/or the drug has adverse effects (*δ* > 0), then RPB will be smaller or even could become negative. In practice, properly quantifying *f*_1_ and *f*_2_ may be very complex. However, if the analyses of the efficacy of the drug for cancer prevention and early detection are only limited to the diseased group (ignore *b* and *d* and *f*_1_≠0) then RPB is equal to the marker’s sensitivity multiplied by $\frac{\left(\eta f_{1}-\delta \right)}{f_{1}}$.

*Example 5: CNV and neuroblastoma*. A copy number variation associated with neuroblastoma was reported recently [15]. The prevalence of the marker (1q21.1) in the general population is about 9%, and the OR of the marker (copy loss) for neuroblastoma risk is estimated to be around 3. If this marker were dichotomized as a binary marker for predicting the absence or presence of the disease, it will result in a 23% sensitivity and 91% specificity, with a PAR% of approximately 15%, which indicates the marker could account for about 15% of neuroblastoma risk if the disease is truly caused by the CNV (copy-number variation). Assume a drug is developed that targeted this marker (1q21.1) for prevention. If the drug is 100% effective in disease prevention and had no side effects and all persons who were carriers for the marker were treated with the drug, it would reduce the total disease cases by 15% (PAR%). However, in the more likely scenario, drugs have significant side effects and are not 100% effective such that more extensive risk benefit analyses are needed. The RPB proposed in this paper could be used for quantifying and evaluating the feasibility for population intervention in such a case.

*Example 6: RPB calculation for three cancers for binary markers or risk factors*. The utility weights for quality-adjusted life years have been estimated for surgical treatment of esophageal adenocarcinoma [16], breast cancer [17], and ovarian cancer [18]; these are 0.97; 0.94; and 0.85 respectively. The corresponding adjustment factors for loss of quality of life (*f*_2_) are 0.03, 0.06, and 0.15 respectively for the three cancers. If a cancer were detected early and intervention were a complete success, this would lead to a benefit value of 1 (true positive detected early); if a subject were wrongly diagnosed with cancer and surgery was done, the cost value can be represented as the loss adjustment factor for quality-adjusted life year. This leads us to have *f*_1_ = 1, *f*_2_ = loss-adjustment factor of a disease intervention to calculate RPB proposed above. Using breast cancer as an example, *f*_2_ = 0.06, since $\mathrm{RPB}=\frac{af_{1}-bf_{2}}{af_{1}+bf_{2}+cf_{1}+df_{2}}=\frac{a\times 1-b\times 0.06}{a\times 1+b\times 0.06+c\times 1+d\times 0.06}$, where *a, b, c,* and *d* are the number of true positive, false positive, false negative, and true negative due to using a biomarker for disease outcome prediction for intervention. In many cases, the OR has been estimated for a marker or risk factors. In Table 1, we show the relationship among RPB and various ORs and prevalence of a marker or risk factor using the loss-adjustment factors of the three cancers as examples. We also calculated net benefit (NB) values under these scenarios for comparison. For instance, from Table 1, if a biomarker has 1% prevalence with OR 10 for breast cancer risk, then the RPB = 0.004, if OR = 20, RPB = 0.014. If a biomarker has 10% prevalence with OR 10, then the RPB = -0.008; if OR = 20, RPB = 0.017. It will be possible to apply these principles to other diseases, novel risk assessments and new treatments as additional data become available. Table 1 shows numerical examples with the assumption of 1% disease prevalence. Disease prevalence of a population will directly affect RPB as the *a* and *c* in the 2×2 table are used in the calculation of RPB. For instance, if disease prevalence is changed from 1% to 3%, and assuming a biomarker (or exposure) prevalence of 30%, the RPB for a risk biomarker with various OR values 1.5, 2, 4, 10, 20, and 50 will be: 0.05, 0.086, 0.172, 0.266, 0.311, 0.345 respectively for EA; -0.064, -0.04, 0.02, 0.084, 0.116, 0.139 respectively for breast cancer; and -0.18, -0.167, -0.134, -0.099, -0.082, -0.069 respectively for ovarian cancer.

#### RPB for continuous markers and risk factors

For a continuous marker, the ‘naïve’ ratio of population benefit for early cancer detection (present or absent) or prevention can be calculated as $\mathrm{RPB}=\frac{wf_{1}\int_{c}^{\infty }f_{d}\left(x\right)\mathrm{dx}-\left(1-w\right)f_{2}\int_{c}^{\infty }f_{\bar{d}}\left(x\right)\mathrm{dx}}{wf_{1}\int_{c}^{\infty }f_{d}\left(x\right)\mathrm{dx}+\left(1-w\right)f_{2}\int_{c}^{\infty }f_{\bar{d}}\left(x\right)\mathrm{dx}+wf_{1}\int_{-\infty }^{c}f_{d}\left(x\right)\mathrm{dx}+\left(1-w\right)f_{2}\int_{-\infty }^{c}f_{\bar{d}}\left(x\right)\mathrm{dx}}$, where *w* is disease prevalence in a population, *f*_*d*_(*x*) and $f_{\bar{d}}\left(x\right)$ are the probability density distribution of a biomarker in the diseased and the non-diseased group respectively and *c* is the cutoff threshold for positive and negative calls for a continuous marker. This formula can also be modified to address potential confounding factors using stratified analysis. The adjusted RPB for a confounding factor of a continuous marker is: $\mathrm{RP}B_{\mathrm{adj}}=\sum_{i}p_{i}\frac{w_{i}{f_{1}}_{i}\int_{c}^{\infty }{f_{d}}_{i}\left(x\right)\mathrm{dx}-\left(1-w_{i}\right)f_{2i}\int_{c}^{\infty }f_{\bar{d}i}\left(x\right)\mathrm{dx}}{w_{i}f_{1i}\int_{c}^{\infty }f_{\mathrm{di}}\left(x\right)\mathrm{dx}+\left(1-w_{i}\right)f_{2i}\int_{c}^{\infty }f_{\bar{d}i}\left(x\right)\mathrm{dx}+w_{i}f_{1i}\int_{-\infty }^{c}f_{\mathrm{di}}\left(x\right)\mathrm{dx}+\left(1-w_{i}\right)f_{2i}\int_{-\infty }^{c}f_{\bar{d}i}\left(x\right)\mathrm{dx}}$, where *p*_*i*_ is the proportion of cases in stratum *i*, and the quantification for loss *f*_1*i*_, and *f*_2*i*_ are the same as in RPB above but are estimated in specific stratum *i.* The density distribution functions *f*_*di*_(x) and $f_{\bar{d}i}\left(x\right)$ are the density distributions of a biomarker in the disease and the non-disease group respectively in stratum *i.*Similar to binary markers, the ‘naïve’ ratio of population benefit for cancer prevention using continuous markers with consideration of adverse effects could be calculated as $\mathrm{RPB}=\frac{wf_{1}\int_{c}^{\infty }\eta \left(x\right)f_{d}\left(x\right)\mathrm{dx}-w\int_{c}^{\infty }\delta \left(x\right)f_{d}\left(x\right)\mathrm{dx}-\left(1-w\right)\int_{c}^{\infty }\delta \left(x\right)f_{\bar{d}}\left(x\right)\mathrm{dx}}{wf_{1}\int_{c}^{\infty }f_{d}\left(x\right)\mathrm{dx}+\left(1-w\right)f_{2}\int_{c}^{\infty }f_{\bar{d}}\left(x\right)\mathrm{dx}+wf_{1}\int_{-\infty }^{c}f_{d}\left(x\right)\mathrm{dx}+\left(1-w\right)f_{2}\int_{-\infty }^{c}f_{\bar{d}}\left(x\right)\mathrm{dx}}$, where *η(x)* is efficacy (as a function of x) of a prevention measure and *δ(x)* represents adverse effect due to a prevention measure. Similar calculations can be used to obtain RPB if *δ(x)* and *η(x)* are dependent on *f*_*d*_(*x*) and $f_{\bar{d}}\left(x\right)$*.* The total lives gained for early cancer detection and cancer prevention using a continuous marker may be quantified as $\left(wf_{1}\int_{c}^{\infty }f_{d}\left(x\right)\mathrm{dx}-\left(1-w\right)f_{2}\int_{c}^{\infty }f_{\bar{d}}\left(x\right)\mathrm{dx})-h(w\int_{c}^{\infty }f_{d}\left(x\right)\mathrm{dx}+\left(1-w\right)\int_{c}^{\infty }f_{\bar{d}}\left(x\right)\mathrm{dx}\right)$, and $\left(wf_{1}\int_{c}^{\infty }\eta \left(x\right)f_{d}\left(x\right)\mathrm{dx}-w\int_{c}^{\infty }\delta \left(x\right)f_{d}\left(x\right)\mathrm{dx}-\left(1-w\right)\int_{c}^{\infty }\delta \left(x\right)f_{\bar{d}}\left(x\right)\mathrm{dx})-h(w\int_{c}^{\infty }f_{d}\left(x\right)\mathrm{dx}+\left(1-w\right)\int_{c}^{\infty }f_{\bar{d}}\left(x\right)\mathrm{dx}\right)$, respectively. As defined for binary markers above, *h* is the coefficient for quantification of the cost of prevention or treatment for exposed subjects or subjects with positive markers; the other parameters remain the same as defined for binary markers. For example, BMI (body mass index) is a continuous marker [19]; studies have shown the association between high BMI and esophageal adenocarcinoma risk [20-22]. If this is a causal association, then BMI may not be a robust marker for detecting presence or absence of disease. However, if BMI were considered as a modifiable risk factor, then false positives may be tolerable since reducing BMI for those people who would never develop esophageal adenocarcinoma had their BMI not been reduced will likely not have substantial detrimental effects (*δ* = 0, RPB always >0). If we use BMI ≥30 as the threshold *c* and assumed no negative effect (*δ* = 0) for reducing BMI for those who have a BMI ≥30, then the $\mathrm{RPB}=\frac{wf_{1}\int_{c}^{\infty }\eta \left(x\right)f_{d}\left(x\right)\mathrm{dx}}{wf_{1}\int_{c}^{\infty }f_{d}\left(x\right)\mathrm{dx}+\left(1-w\right)f_{2}\int_{c}^{\infty }f_{\bar{d}}\left(x\right)\mathrm{dx}+wf_{1}\int_{-\infty }^{c}f_{d}\left(x\right)\mathrm{dx}+\left(1-w\right)f_{2}\int_{-\infty }^{c}f_{\bar{d}}\left(x\right)\mathrm{dx}}$; where *η(x)* is the efficacy of cancer reduction by reducing BMI, *f*_*d*_(*x*) and $f_{\bar{d}}\left(x\right)$ are the BMI distribution in a specific at risk population and low risk population, respectively. However, if negative effects occur when reducing BMI (*δ* > 0), e.g., if a medication used for weight loss is associated with significant side effects, then the RPB will be smaller. The RPB, therefore, could be used to quantify the potential overall benefit of a prevention measure targeted to a marker or risk factor. Similar to binary markers, if the analysis of effects for prevention and disease detection is restricted to the diseased group only (ignore $f_{\bar{d}}\left(x\right)$ in RPB above) for a continuous marker in prevention, and assuming no negative effect (*δ* = 0), then the RPB becomes $\frac{\int_{c}^{\infty }\eta \left(x\right)f_{d}\left(x\right)\mathrm{dx}}{\int_{c}^{\infty }f_{d}\left(x\right)\mathrm{dx}+\int_{-\infty }^{c}f_{d}\left(x\right)\mathrm{dx}}$ , which is an estimation of prevention effects with consideration of at risk subjects only, and it will always have a positive value (benefit).

*Example 7: RPB calculation for continuous markers or risk factors*. To evaluate the benefit of using a continuous biomarker or risk factor for disease intervention, the probability density distribution of the marker in the population of disease outcome *f*_*d*_(*x*) and non-disease outcome $f_{\bar{d}}\left(x\right)$ can be estimated from observed population data. Then, to calculate RPB of the continuous biomarker, a threshold *c* of the biomarker is chosen (i.e., any subjects with the biomarker level above the threshold will be predicted to have the disease outcome); then numerical integration can be used to obtain RPB for the continuous markers using the formula above. For example, using the same loss of quality of life adjustment factors of the three cancers shown in Example 6 (the loss factor *f*_*2*_ are 0.03, 0.06, and 0.15 for the three cancers), assume the probability distributions of a continuous marker in the population with disease *f*_*d*_(*x*) and without disease $f_{\bar{d}}\left(x\right)$ follow the normal distributions with different means as shown in Figure 1. Using numerical integration for the RPB formula above, we calculated the RPB for various scenarios in which the hypothetical continuous markers have different OR for the cancer risk as shown in Table 2. In these calculations, the thresholds *c* of the continuous biomarker were chosen to demonstrate various possibilities for outcome prediction including fixed sensitivity, fixed specificity, and balanced sensitivity and specificity. Table 2 shows numerical examples with assumption of 1% disease prevalence for RPB calculation. Disease prevalence *w* of a population will directly affect RPB value. For example, for a breast cancer biomarker with balanced sensitivity and specificity if the disease prevalence is changed from 1% to 3%, the corresponding RPB values for a risk biomarker with various OR values 1.5, 2, 4, 10, 20, and 50 will be changed from -0.316, -0.285,-0.222, -0.137, -0.085, -0.020 for 1% prevalence to -0.12, -0.09, -0.025, 0.058, 0.112, 0.176 respectively for 3% prevalence.

## Discussion

With rapid advances in various technologies, a large numbers of biomarkers have been reported to be associated with various diseases, including cancer. Translating those to the clinic and for public health benefit is a critical but difficult next step. For example, genome-wide association studies are identifying hundreds of SNPs associated with a variety of diseases. While the rich discoveries from such studies continue to prompt investigation of pathway-targeted interventions for disease prevention and therapy [6], it is generally believed that use of single or combined SNP information can achieve only modest improvement in disease risk prediction or early detection programs for individuals in the general population as compared to current clinical screening modalities [23-27]. This underscores the need for quantitative evaluation of both the risk and potential benefit of biomarkers since in this process many factors need to be considered including sensitivity and specificity of the marker used for risk prediction or targeted therapy, disease prevalence and quantitative relationship between biomarker levels and meaningful risk measures, cost, and risk/benefit analyses [28-30]. In this paper, we call attention to the need to distinguish and quantify the consequences of false positive and false negative diagnoses of a marker for prevention and early detection/risk prediction. The RPB estimation can be confounded due to the marker or risk exposure. Adjustment for potential confounders in estimating RPB need be considered.

Table 1 shows the numerical relationships between measurements commonly used for cancer detection and prevention. For a marker with a low prevalence, the sensitivity of the marker is very low even when it has a high OR value. Combining multiple SNP/CNV markers with low OR values for prediction may have limited effects at the population level, i.e. a person who carries all 10 ‘risk’ SNPs will be at high risk for the disease; however very few people carry all 10 SNPs in the general population, thus leading to a low PAR% value. Therefore, using such a panel may have a low impact on disease detection or risk prediction in the general population, although it might be applied to the few individuals in that category. Based on three published data sets, the numerical calculations of RPB for the three cancers are presented in Tables 1 and 2. The calculations show that due to low specificity of a marker and the disease intervention action based on the prediction of the markers in the tables, risk is larger than benefit (RPB < 0) in many cases. However, we used the loss of quality of life adjustment factor from the three cancers in a simple way to demonstrate the application of the RPB. The risk and benefit may be affected by many factors such as age, time, or unknown confounders.

Six phases are recommended for developing effective biomarkers, with longitudinal studies considered essential for validation [31]. Pepe *et al*. [32] presented a comprehensive method for evaluating the predictiveness of a biomarker and its performance as a disease classifier. The application of PAR% was evaluated for benefit of community-based efforts to prevent disease using a specific cancer marker by Wacholder [33]. Furthermore, the quantitative connection between biomarker levels in cases and controls and clinical meaningful risk measures or testing also has been carefully evaluated by Wentzensen and Wacholder [30], adding a useful tool for apprising candidate biomarkers at an early stage. The RPB proposed in this study takes account of both accuracy of outcome prediction of a marker and benefit for a population if the marker were used for an intervention. The prediction accuracy of biomarker(s) should be assessed and compared with validated or well-established tools (such as area under the curve, integrated discrimination improvement, net reclassification improvement etc.). Then the value of biomarker(s) should be further evaluated for risk and benefit if it were to be applied in a large population for intervention. In this paper, we assumed the selection of biomarker(s) for prediction has been completed, and we propose the RPB for risk and benefit analysis at the population level when a given marker or risk factor is used for disease intervention. Specifically, we concentrate on the framework of risk/benefit analysis in using a marker for disease prevention and detection/prediction.

The 1,000 Genomes Project is expected to discover substantially more SNP markers and other variants that have frequency between 0.5 to 5%. Those data could be analyzed by the methods presented in this paper. Thus far, the performance of SNP/CNV for disease risk prediction or risk stratification still needs improvement [34], while their potential for disease prevention or targeted therapy [7] and prediction of prognosis [35] is substantially encouraging. A broad risk/benefit analysis will be needed when translating the results of such studies into clinical use for cancer risk prediction, detection and prevention. Greenland [36] pointed out that the evaluation of marker prediction models are linked to predictor-conditional performance, cut-point choices, and error costs; and there is a need for reorientation toward cost-effective prediction. We extended the issue further by distinguishing between using biomarkers for early disease detection and using them for prevention. The feasibility or value of using biomarkers in the two scenarios could be generalized by risk/benefit analysis, a research direction that has been proposed in previous studies [37,38]. The proposed RBP for binary and continuous markers/exposures can be extended to health economics studies.

The National Cancer Institute recently identified challenges for cancer epidemiology in the 21st century [39]. Eight overarching recommendations with corresponding actions were proposed by the scientific community for consideration [40]. Here, we propose methods that can be used for assessing risk and benefit of disease intervention based on a biomarkers or risk factors, which are particularly pertinent to two of the eight recommendations: (1) “balance the epidemiology research portfolio beyond traditional emphasis on discovery and etiology research to encompass development and evaluation of clinical and population interventions, implementation, dissemination, and outcomes research”; and (2) “support knowledge integration and meta research (systematic reviews, modeling, decision analysis etc.) to identify gaps, inform funding, and to integrate epidemiologic knowledge into decision making”. The RPB is intended to illustrate an approach to assess the risk and potential benefits using a marker/risk factor for intervention in both early detection and prevention. As such, it is a general framework, and requires a proper estimation of ‘risk/benefit’ quantifications (*f*_1_ and *f*_2_) in the RPB model for each disease. Substantial effort may be needed to properly estimate such parameters for a biomarker to properly evaluate the feasibility of using the biomarkers for different scenarios such as early detection, risk prediction, and prevention.

## Conclusions

Making use of the discovered biomarkers/risk factors from epidemiological and clinical research for clinical decision making is a key step to translate the discoveries into practical use to achieve health benefits. Risk benefit analysis provides crucial information for disease intervention decision making. It is worthwhile to distinguish and quantify the differences between the use of biomarkers/risk factors to identify preventive interventions versus their use in disease risk prediction and early detection. The RPB proposed in this paper not only considers the absolute risk of a disease in intervention, but also takes into account risk-benefit effects simultaneously for a marker/exposure at the population level. Using concrete examples, we demonstrate that RPB developed in this study is a useful tool for quantitatively assessing the risk and benefits in using a biomarker/risk factor for intervention in both early detection and prevention.

## Abbreviations

CNV: (somatic) Copy number variation; OR: Odds ratio; PAR%: Population attributable risk percent; RPB: The ‘naïve’ Ratio of population benefit; SNP: Single nucleotide polymorphism.

## Competing interests

All authors of this paper declare that they have no competing interests.

## Authors' contributions

Author XL initiated the prototype of the manuscript in the environment of close working relationships with other co-authors of the manuscript. PLB and BJR provided input from the clinical application point of view. TLV provided input from the epidemiology and population science point of view. All authors were involved in vigorous discussions during the writing of the manuscript. XL performed the computer programming for all numerical calculations. All authors of the manuscript were actively involved in the editing and finalization of the submitted manuscript. All authors read and approved the final manuscript.

## Pre-publication history

The pre-publication history for this paper can be accessed here:

http://www.biomedcentral.com/1472-6947/14/15/prepub
